# The Sexual Risk Behaviors Scale (SRBS): Development & Validation in a University Student Sample in the UK

**DOI:** 10.1177/01632787211003950

**Published:** 2021-04-15

**Authors:** Emanuele Fino, Rusi Jaspal, Bárbara Lopes, Liam Wignall, Claire Bloxsom

**Affiliations:** 1 Department of Psychology, 6122 Nottingham Trent University, United Kingdom; 2 Center for Research in Neuropsychology and Cognitive and Behavioral Intervention (CINEICC), 37829 Universidade de Coimbra, Coimbra, Portugal; 3 6657 Bournemouth University, Poole, United Kingdom

**Keywords:** University students, sexual health, sexual risk, sexually transmitted infections, scale validation

## Abstract

University students are at risk of poor sexual health outcomes. The aim of this study was to develop and test the psychometric properties of the Sexual Risk Behaviors Scale (SRBS), a novel short tool for measuring engagement in sexual risk behaviors in university students. We developed a pool of six items based on a review of recent literature and tested its properties in 547 undergraduate students in the United Kingdom. We used Exploratory Factor Analysis and Confirmatory Factor Analysis to explore and determine the factor structure and dimensionality of the SRBS. We used Item Response Theory and specifically the Graded Response Model to investigate items’ discrimination, information, and differential functioning, respectively, and logistic regression to test whether higher SRBS scores predicted a diagnosis of any sexually transmitted infections in the past 12 months. Results showed that a unidimensional, five-item model fitted the data well, showing satisfactory fit indices and reliability, with all items providing adequate discrimination and information, and no differential item functioning by gender nor by sexual orientation. SRBS total scores significantly predicted the odds of being diagnosed with sexually transmitted infections in the past 12 months. Implications for public health prevention and intervention are discussed.

## Introduction

Students face significant identity changes as they embark upon university life (Fino et al., 2018, 2020; Jaspal, 2020). These include leaving their families and households, adapting to a new environmental context, creating new relationships and networks, and developing their sexual identities (Patton et al., 2016). Research has shown that university students tend to report high rates of sexual risk behaviors, often leading to negative health outcomes, to the point of being recently described as “a key population in sexual health epidemiology” (Jaspal, 2020, p. 159). To assess sexual risk in this key population, a short, valid, and reliable measure of sexual risk is necessary and is therefore proposed in this article.

Recent systematic reviews of the literature suggest that a unique definition of sexual risk behaviors is missing (Chawla & Sarkar, 2019; Mirzaei et al., 2016). However, several studies refer to sexual risk behaviors as increasing an individual’s likelihood of developing negative health and wellbeing outcomes (Chanakira et al., 2014; Chawla & Sarkar, 2019; Jaspal, 2020; Jaspal et al., 2021; Mirzaei et al., 2016; Vivancos et al., 2010), including sexually transmitted infections (STIs), unwanted pregnancies, conflictual relationships with family, friends, and/or partners, and legal and financial issues. Research on sexual risk behaviors in university students has focused on unprotected casual sex (Bailey et al., 2011), unprotected anal sex (Luo et al., 2020), inconsistent condom use (Pinyaphong et al., 2018; Rodrigues Moreira et al., 2018), and sex under the effects of alcohol (White & Hingsom, 2013) and other substances (Vivancos et al., 2008, 2010). These behaviors are of significant clinical interest due to their association with negative health outcomes (Chanakira et al., 2014). Inconsistent condom use in anal intercourse is of particular concern, as it is associated with a high likelihood of STIs’ transmission, including HIV (Luo et al., 2020). Moreover, it is acknowledged that other types of sexual behavior, such as “kink” and bondage, discipline, dominance and submission, and sadomasochism (BDSM), may be related to engagement in sexual risk in some populations (McGregor, 2015). However, to the best of our knowledge, evidence that these behaviors are prevalent in university students is limited (De Neef et al., 2019). Thus, it might be sensible to conceptualise them as correlates of sexual risk behaviors, rather than constituting sexual risk behaviors per se, in university students. Moreover, there is evidence that students may be more likely to endorse sexual risk behaviors under specific activating conditions, such as when being sexually aroused (Ariely & Loewenstein, 2006).

Considering that about two and a half million individuals were enrolled in UK higher education institutions in the academic year 2018/19 (Bolton, 2020), effective assessment of sexual risk behaviors in this population represents a public health priority. Although there are no specific data available on the sexual health of university students in the UK, it is known that the incidence of STIs among young people (aged 15–24) in the UK is high, and that most undergraduate university students fall within this age group (Department of Education, 2019). Latest official public health records from England showed 420,000 STIs diagnoses in 2017, about 34% of which occurred among young people (Public Health England, 2018). However, there is also evidence that most young people in England do not regularly screen for STIs and that they are likely to be living with undiagnosed infections, which may in turn lead to risk for peer-transmission and long-term health conditions (Public Health England, 2018).

Identifying university students who are at risk of poor sexual health is therefore key to the development and implementation of effective prevention and intervention programs, and to reducing STIs rates and negative short- and long-term physical, mental, and sexual health outcomes in this population (Turchik & Garske, 2009). In this regard, Mirzaei et al. (2016) highlighted the importance of developing valid and reliable instruments for assessing risk behaviors to promote health and wellbeing outcomes. However, recent research shows that university students face several barriers to accessing sexual health services (Bender & Fulbright, 2013), being often required to miss class and/or to modify their schedules to attend sexual health services, which in turn, reduces the likelihood of receiving adequate assessment and treatment (Cassidy et al., 2018). A short tool for measuring engagement in sexual risk behaviors would enable both researchers and clinicians to identify those at risk. This would represent a significant public health asset to tackle sexual risk behaviors in university students, ensuring early detection of those at risk of poor sexual health outcomes.

Psychometric scales assessing sexual risk behaviors exist, and they were classified by Mirzaei et al. (2016) in two main categories: (i) questionnaires assessing a variety of sexual risk behaviors and predisposing factors, and (ii) questionnaires assessing specific risk factors for STIs, or other health outcomes (e.g., condom use). The following scales belong to the first category: The Sexual Risk Survey (Turchik & Garske, 2009; Cronbach’s α of factors = 0.61–0.93), the Safe Sex Behavior Questionnaire (Dilorio et al., 1992; Cronbach’s α of factors = 0.52–0.85), the Sexual Health Practices Self-Efficacy Scale (Koch et al., 2013; Cronbach’s α of factors = 0.71–0.82) and the Sexual Risk Behavior Beliefs and Self Efficacy Scales (Basen-Engquist et al., 2013; Cronbach’s α of factors = 61–87). Each of these scales underlies a multifactorial model (Mirzaei et al., 2016).

As sexual behavioral patterns are changing, there is a need to develop tools that would enable us to capture this change, as well as novel behaviors that may be becoming more common in the university student population. Drawing upon qualitative research into student sexual behavior and health in the UK (Jaspal, 2020), we propose a short measure of frequency of engagement in key sexual risk behaviors reported among UK university students, with a strong focus on sex with casual partners and the use of alcohol and substances before or during sex. The proposed measure shifts the exclusive focus on condomless sex which has characterized several previously published scales of sexual risk. There is also a shift from the focus on the number of actual sexual partners to the frequency of particular sexual risk acts (such as, but not limited to, engaging in condomless anal sex). Moreover, in contrast to existent scales, which focus on related psychological constructs such as beliefs and self-efficacy in relation to sexual behaviors, there is a need for robust measures of frequency of engagement in sexual risk behaviors.

Crucially, the short scale that we propose examines the subjective frequency of engaging in such behaviors which provides a more general inclination to take sexual risks. This is important partly because short, reliable and valid assessments of sexual risk behaviors are lacking, despite their great potential utility to target the university student population for an effective first line assessment and primary care. Furthermore, when used in conjunction with more “objective” measures of sexual risk, the proposed scale may help shed light on the relationship between objective risk and perceived frequency of engagement in sexual risk behavior, thereby facilitating, for the health professions in particular, a tool for building awareness and understanding of risk in student populations. For this reason, in the current study, we sought to develop and test the psychometric properties of the Sexual Risk Behavior Scale (SRBS), a short psychometric scale that could provide institutions and practitioners with a reliable, valid, and efficient assessment of sexual risk behaviors in university students.

We followed recommendations from the literature to develop and test a pool of items whose content could maximize the fit to the measurement objectives derived from contemporary literature on sexual risk behaviors, using a language that could be easily understood by individuals from a university student population, and an adequate format to collect responses (DeVellis, 2016). In the following paragraphs, we present the results of the development, exploration, and testing of the psychometric properties of the SRBS in two UK university samples, with a focus on its factor structure, reliability, item functioning, and validity.

## Methods

We conducted a prospective psychometric scale development and validation study in three phases: (i) scale development, (ii) content validation, and (iii) psychometric testing.

### Phase 1: Scale Development

We started developing the SRBS based on a of review of recent research on sexual risk behaviors as major determinants of poor sexual health outcomes in university students (Chanakira et al., 2014; Jaspal, 2019, 2020; Jaspal et al., 2021; Lewis et al., 2017; Mirzaei et al., 2016; Vivancos et al., 2008, 2010). This process led us to identify several key sexual risk behaviors, for which we formulated and developed a pool of 13 items to measure them. For all items, we devised a response format based on a 5-point Likert scale, measuring the frequency with which students had engaged in those sexual risk behaviors in the past month (0 = “Never”; 1 = “Rarely”; 2 = “Sometimes”; 3 = “Often”; 4 = “Very often”).

### Phase 2: Content Validation

To determine the content validity of the scale, we asked a panel of three experts in psychology and human sexuality to read and critique the newly developed items, particularly the extent to which they effectively measured relevant sexual risk behaviors, taking into account three main criteria: (i) their relevance to poor sexual health outcomes, (ii) the clarity and adequacy of wording, and (iii) their suitability to and significance for the target population. We also asked each panel member to provide recommendations with regard to any items that might require changes or modifications. Eventually, panelists confirmed that six out of the original 13 items satisfied the three criteria. The excluded items were evaluated to elicit a range of students’ memories, experiences, and perceptions tackling correlates of sexual risk behaviors (e.g., commercial sex, sexual abuse, discussing contraception with partners, and regret associated with sexual risk behavior), rather than measuring the frequency of sexual risk behaviors per se, ultimately failing to satisfy the criterion of coherent elaboration that is necessary for establishing cognitive validity (Karabenick et al., 2007; see also Elliot, 2005). For this reason, we proceeded to the psychometric testing of the six-item version of the SRBS.

### Phase 3: Psychometric Testing

#### Participants and procedure

We contacted a population of undergraduate students on two UK university campuses, one in the East Midlands region and the other in the South of England region, respectively. Students were informed that this was a study on sexual behavior, sexual risk and sexual health, aiming to enhance the measurement of sexual risk behaviors in student populations. They were also informed that participation was voluntary and that refusal to participate involved no penalties. The inclusion criteria were: (i) to be aged at least 18–25 years, (ii) to be registered at a UK university as an undergraduate student, and (iii) to be able to read and understand English to a sufficient level to complete the procedure.

Those who expressed interest were invited to read, accept, and sign an informed written consent form. Finally, 551 students were recruited and completed the study procedure. Participants were aged 18–25 (*M* = 19.95, *SD* = 1.56), of whom 376 (68.24%) self-reported to be female, 172 male (31.22%), and 3 (0.54%) of non-binary gender. They came from various ethnic and religious backgrounds. Table 1 provides details of the participants’ socio-demographic characteristics.

**Table 1.** Social and Demographic Characteristics of Participants.

Variables	Total (*N* = 551)	Females (*n* = 376)	Males (*n* = 172)	Non-Binary (*n* = 3)	*p^a^*
**Age (years)** *M* (*SD*)	19.9 (1.6)	19.6 (1.3)	20.7 (1.9)	20.7 (1.2)	0.000
**Orientation** *N* (%)					0.000
Heterosexual	423 (76.77%)	287 (76.33%)	136 (79.07%)	0 (0%)	
Gay	17 (3.09%)	2 (0.53%)	15 (8.72%)	0 (0%)	
Lesbian	10 (1.81%)	9 (2.39%)	0 (0%)	1 (33.33%)	
Bisexual	92 (16.7%)	71 (18.88%)	19 (11.05%)	2 (66.67%)	
Other	9 (1.63%)	7 (1.86%)	2 (1.16%)	0 (0%)	
**Relationship Status** *N* (%)					0.070
Single	329 (59.71%)	212 (56.38%)	116 (67.44%)	1 (33.33%)	
Monogamous relationship	191 (34.66%)	146 (38.83%)	43 (25%)	2 (66.67%)	
Open relationship	4 (0.73%)	3 (0.8%)	1 (0.58%)	0 (0%)	
Engaged	12 (2.18%)	5 (1.33%)	7 (4.07%)	0 (0%)	
Married	2 (0.36%)	2 (0.53%)	0 (0%)	0 (0%)	
Civil partnership	6 (1.09%)	2 (0.53%)	4 (2.33%)	0 (0%)	
Other	7 (1.27%)	6 (1.6%)	1 (0.58%)	0 (0%)	
**Ethnicity** *N* (%)					0.000
White British	327 (59.35%)	252 (67.02%)	72 (41.86%)	3 (100%)	
Mixed	132 (23.96%)	65 (17.29%)	67 (38.95%)	0 (0%)	
Asian	48 (8.71%)	26 (6.91%)	22 (12.79%)	0 (0%)	
Black, African, Caribbean	37 (6.72%)	29 (7.71%)	8 (4.65%)	0 (0%)	
Other	7 (1.27%)	4 (1.06%)	3 (1.74%)	0 (0%)	
**Religion** *N* (%)					0.000
No religion	389 (70.6%)	263 (69.95%)	123 (71.51%)	3 (100%)	
Muslim	20 (3.63%)	14 (3.72%)	6 (3.49%)	0 (0%)	
Buddhist	6 (1.09%)	0 (0%)	6 (3.49%)	0 (0%)	
Hindu	15 (2.72%)	5 (1.33%)	10 (5.81%)	0 (0%)	
Jewish	3 (0.54%)	2 (0.53%)	1 (0.58%)	0 (0%)	
Sikh	14 (2.54%)	4 (1.06%)	10 (5.81%)	0 (0%)	
Christian	91 (16.52%)	79 (21.01%)	12 (6.98%)	0 (0%)	
Other	13 (2.36%)	9 (2.39%)	4 (2.33%)	0 (0%)	

^a^ Results from parametric bivariate tests of significance (ANOVA for continuous variables and *χ*^2^ test of independence for categorical variables).

#### Measures

The Sexual Risk Behavior Scale (SRBS) was originally composed of six items, each measuring a specific sexual risk behavior. All items were scored on a 5-point Likert scale. Items’ descriptors included: “How often have you had vaginal sex without a condom?”, “How often have you had anal sex without a condom?” “How often have you performed oral sex without protection (condom or dental dam)?” “How often have you had sex while under the influence of alcohol (i.e. drunk)?” “How often have you had sex while under the influence of drugs or substances?” “How often have you had sex without a condom with someone you have just met?”

Additionally, we asked participants to indicate whether they had been diagnosed with any STIs, in the past 12 months (“Yes,” “No).

#### Statistical analyses

To explore the dimensionality and factor structure of the SRBS, we used Exploratory Factor Analysis (EFA). We subsequently used Confirmatory Factor Analysis (CFA) to test the theoretical model derived from EFA, and Item Response Theory (IRT) to evaluate item discrimination, Item Information Function (IIF), and Differential Item Functioning (DIF).

EFA is a statistical technique that examines the structure of latent variables (factors) underlying a set of observed variables (items). The starting point of EFA is the correlation matrix of observed variables and the endpoint is the correlation matrix between observed variables and latent factors (Costello & Osborne, 2005). Considering the ordinal nature of the data, we used polychoric correlations and ran EFA using the Weighted Least Square (WLS) estimation method (Schmitt, 2011). We used three criteria to evaluate the factor structure and the dimensionality of the SRBS: (i) parallel analysis (Horn, 1965), (ii) the very simple structure method (Revelle & Rocklin, 1979), and (iii) the theoretical interpretability and internal consistency of the factor solution.

Parallel analysis is a technique that compares the eigenvalues obtained from the empirical correlation matrix to the eigenvalues obtained from simulated data sets of equal size. The empirical eigenvalues that result greater than their simulated counterparts indicate the best candidates for factor retention. The very simple structure method compares alternative factor solutions obtained by progressively constraining factor loadings to zero, except for the top-loading item. The solution that maximizes the fit to the empirical correlation matrix represents the best candidate for retention.

CFA is a statistical technique that tests a theoretical model of latent variables on a set of observed variables, enabling the researcher to evaluate the fit of the theoretical model to the data. The goodness of the fit can be assessed by means of specific indices (Brown, 2006). We used the following indices and criteria (Kenny, 2015): The Comparative Fit Index (CFI) ≥ 0.95, the Root Mean Square Error of Approximation (RMSEA) ≤ 0.07, and the Standardized Root Mean Square Residual (SRMR) ≤ 0.08. We ran CFA with robust weighted least squared estimation (WLSMV), using the diagonal of the weight matrix to determine the model and the full weight matrix to estimate test statistics and robust standard errors (Brown, 2006; Rosseel, 2012). The internal consistency and reliability of the SRBS were assessed by using Cronbach’s α and McDonald’s (1999) Omega, respectively. In particular, we used Green and Yang’s (2009) Omega formula 21, accounting for item covariances and item thresholds, as required for ordinal data.

We used IRT to estimate items’ discrimination, IIF, and DIF by gender and sexual orientation. IRT is a family of statistical models that rely on the fundamental assumption that item responses depend on the respondent’s location onto a hypothesized latent dimension (*θ*) (van der Linden & Hambleton, 1997). We used the Graded Response Model (GRM) to estimate items’ discrimination, i.e. their ability to differentiate students by their frequency of engagement in sexual risk behaviors, and the statistical information accounted for by each item (Samejima, 1969). The GRM assumes that the probability of endorsing a higher response option increases proportionally to the respondent’s level of the assumed latent dimension. Items’ threshold parameters indicate the point on the latent dimension in which a respondent has a 0.50 probability of selecting a specific response option. IIF represented the statistical information over sexual risk behavior that each item accounted for. Prior to fitting the model, we assessed the assumption of dimensionality by examining the results from EFA and CFA, and the assumption of items’ conditional independence by evaluating residual correlations (Linacre, 2009). We used DIF with ordinal logistic regression and χ^2^ (α= 0.01) as detection criterion (Choi et al., 2011).

Finally, we examined the criterion (postdictive) validity of the SRBS by fitting a binomial logistic regression model, aiming to test the hypothesis that SRBS total scores, obtained by averaging across individual items’ scores, significantly predicted any STIs diagnosis in the past 12 months. We used the area under the Receiver Operating Curve (ROC) to evaluate the accuracy of the prediction.

We performed the analyses by using the statistical programming language R (Version 3.6.2) (R Core Team, 2019), and specifically, the following packages: psych (Revelle, 2020) for EFA, lavaan (Rosseel, 2012) for CFA, semTools (Jorgensen et al., 2020) for reliability, semPlot (Epskamp, 2015) for CFA plots, mirt (Chalmers, 2012) for IRT, lordif for DIF (Choi et al., 2011), and furniture for tables (Barret & Brignone, 2017). We randomly extracted two sub-samples from the total sample, with similar sizes and socio-demographic characteristics, and we used the first sub-sample to explore the factor structure and dimensionality of the SRBS and the second sub-sample to test the theoretical model and items’ performance by using CFA and IRT, respectively. Routine data screening, SRBS’ descriptive statistics and correlations, and validity analyses were performed on the total sample.

## Results

Four observations were removed from the dataset due to missing information. All subsequent analyses were run on a total sample of 547 students. Table 2 presents SRBS descriptive statistics and the SRBS item correlation matrix.

**Table 2.** Correlation Matrix (*N* = 547).

Item	*M*	*SD*	Skewness	Kurtosis	1	2	3	4	5
Item 1	1.80	1.63	0.16	−1.62					
Item 2	0.38	0.90	2.62	6.40	0.30***				
Item 3	2.31	1.60	−0.39	−1.44	0.58***	0.31***			
Item 4	1.40	1.22	0.39	−0.90	0.45***	0.24***	0.44***		
Item 5	0.56	1.02	1.77	2.17	0.33***	0.19***	0.27***	0.49***	
Item 6	0.52	0.96	1.93	2.97	0.35***	0.28***	0.29***	0.38***	0.35***

*Note.* *M* and *SD* represent mean and standard deviation, respectively. All correlations are expressed as Spearman’s ρ values.

*** indicates *p* < 0.001.

Parallel analysis conducted on the polychoric correlation matrix from the first sub-sample (*N* = 274) extracted a total of six factors, of which, only the first two displayed empirical eigenvalues (2.86, 0.34, 0.12, −0.07, −0.16, −0.22) greater than the eigenvalues obtained from the simulated data (0.54, 0.30, 0.22, 0.12, 0.04, −0.03). However, the very simple structure method showed that the one-factor model represented the best solution (VSS = 0.73). Further examination showed that the two-factor model presented low internal consistency on both factors (Cronbach’s α of factors = 0.66, 0.68) and multiple cross-loading items (Table 3), whereas the one-factor model presented higher internal consistency (Cronbach’s α = 0.76), with all items loading highly (0.58–0.73) onto the single factor, overall explaining the 48% of the total variance. Based on the results from the exploratory analyses, we retained the unidimensional model for further inspection.

**Table 3.** Exploratory Factor Analysis (*N* = 274).

Item Number	One-Factor Solution			Two-Factor Solution			
	F1	H2	U2	F1	F2	H2	U2
Item 1	0.73	0.53	0.47	0.74	0.05	0.59	0.41
Item 2	0.58	0.34	0.66	0.60	0.01	0.37	0.63
Item 3	0.78	0.61	0.39	0.90	−0.04	0.77	0.23
Item 4	0.72	0.52	0.48	0.30	0.53	0.55	0.45
Item 5	0.64	0.41	0.59	−0.05	0.93	0.82	0.18
Item 6	0.67	0.45	0.55	0.36	0.39	0.44	0.56
Cronbach’s α	0.76			0.66	0.68		
Total variance explained	48%			35%	34%		

*Note.* H2 and U2 represent items’ communalities and individual variances, respectively.

We ran CFA on the second randomly generated sub-sample (*N* = 273), testing a model in which all items loaded onto a single factor. Fit indices indicated an unsatisfactory fit: CFI = 0.961, RMSEA = 0.116, and SRMR = 0.069. Modification indices showed that by releasing constrained error covariances between Item 1 and the other items, the model fit would improve. We re-tested the model after dropping Item 1, finding a better fit (CFI = 0.993, RMSEA = 0.051, SRMR = 0.051), with the solution being internally consistent and reliable (Cronbach’s α = 0.84; Omega = 0.82). For those reasons, we retained the last 5-item model for further testing.

In particular, we fitted and compared two alternative GRM models: (i) a model with unconstrained parameters (Model 1), and (ii) a model in which parameters were constrained to be equal for all items (Model 2) (Rizopoulos, 2006). Results showed that Model 1 (AIC = 2,851.13, BIC = 2,926.93, logLik = −1,404.56, marginal reliability = 0.76) had better fit (χ^2^_(4)_ = 22.57, *p* < .001) to the data compared to Model 2 (AIC = 2,836.56, BIC = 2,926.80, logLik = −1,393.28, marginal reliability = 0.73). The residual correlation matrix from Model 1 showed an absolute average residual correlation of 0.04 and negative residual correlations greater than the estimated absolute critical value (0.24) for Item 4, suggesting local dependence. Moreover, when inspecting items’ parameters, we found that a few response categories for items 2, 3, and 5 were redundant and could be collapsed to improve the statistical and substantive validity of the model (Linacre & Wright, 1992). We proceeded by collapsing the response categories 1 (“Rarely”) and 2 (“Sometimes”) into a single category for Item 2, Item 3, Item 5, and additionally, the response categories 3 (“Often”) and 4 (“Very often”) into a single category for Item 2. We re-tested the unconstrained GRM after collapsing those categories (Model 3) and results showed satisfactory fit (AIC = 2,619.40, BIC = 2,695.20, logLik = −1,288.70, marginal reliability = 0.76). Table 4 reports item parameters from Model 3.

**Table 4.** Graded Response Model, Item Parameters and Standard Errors (*N* = 273).

	*α*	SE	*β*1	SE	*β*2	SE	*β*3	SE	*β*4	SE
Item 2	1.04	0.24	−1.79	0.22	−3.62	0.36				
Item 3	1.24	0.19	1.2	0.18	0.09	0.16	−1.02	0.17		
Item 4	3.14	0.56	1.49	0.33	−0.43	0.28	−2.94	0.48	−5.33	0.76
Item 5	2.39	0.42	−1.56	0.30	−3.87	0.50	−5.95	0.77		
Item 6	1.74	0.42	−1.49	0.30	−2.58	0.50	−3.64	0.77	−5.51	0.42

*Note.* The number of category threshold parameters (*β*) and relevant standard errors (SE) varied after collapsing redundant categories.

Item 4 was the most discriminative item (α = 3.14) and accounted for the greatest amount of IIF (92.08), followed by Item 5 (α = 2.39, IIF = 24.9) and Item 6 (α = 1.74, IIF = 35.94), whereas Item 3 (α = 1.24, IIF = 20.71), and Item 2 (α = 1.04, IIF = 12.44) were the least discriminating items.

We used Model 3 to test for DIF by gender (female vs. male) and sexual orientation (heterosexual vs. non-heterosexual). The results from the regression models showed no DIF, indicating invariance by gender and by sexual orientation across all the SRBS items.

Finally, we evaluated the criterion validity of the SRBS by fitting a generalized linear model, aiming to test whether SRBS total scores significantly predicted any STI diagnosis in the past 12 months. The model was statistically significant (logLik_(2)_ = −96.91; χ^2^_(1)_ = 15.23; *p* < .001), indicating that for a one-unit increase in SRBS scores, the predicted odds of STI diagnosis in the past 12 months increased by about three times (OR = 3.03, 95% CI 1.75–5.30, *p* < .001). The area under the ROC curve was equal to 0.71 (DeLong 95% CI = 0.60–0.82), showing an acceptable accuracy of the classification.

The final version of the SRBS is provided in the Supplemental Appendix.

## Discussion

The aim of the present study was to develop a short, reliable, and valid scale for the assessment of sexual risk behaviors in the university student population. We developed, tested, and found evidence for the reliability and validity of the Sexual Risk Behavior Scale (SRBS) based on a review of evidence from recent literature on sexual risk behaviors. We developed and refined a pool of items to measure frequency of sexual risk behaviors in university students, and we used EFA and CFA to explore and to evaluate our measurement model, respectively.

Results showed that a unidimensional five-item model fits the data well, with all fit indices being acceptable, the scale being internally consistent and reliable, and all items loading highly and significantly onto the factor. We fitted an unconstrained GRM model and found few item response categories that were redundant, and for this reason, we decided to collapse them and to re-test the model. The final model showed satisfactory fit and no differential item functioning by gender nor by sexual orientation, with all items achieving a satisfactory level of discrimination. Regarding the criterion validity of the SRBS, we fitted a generalized linear model to test whether SRBS total scores predicted any STI diagnosis in the past 12 months. Results showed that for a one-unit increase in SRBS scores, the predicted odds of STI diagnosis in the past 12 months increased by about three times. Although this is an important indicator of the validity of the SRBS, future research will need to further examine the validity of the SRBS, particularly by examining its correlation with other relevant constructs, such as attitudes toward condom use, perceived sexual norms, and the acceptability of sex under the influence of alcohol and other substances (Basen-Engquist et al., 2013). These additional indicators will be especially important in view of the relatively low STI testing rates in university students.

The SRBS focuses on key sexual risk behaviors in university students, namely unprotected sex with casual partners, anal and oral sex (as specific high-risk behaviors), sex while under the influence of alcohol, and sex while under the influence of drugs or substances. Despite the brevity of the SRBS, the scale captures a comprehensive range of sexual risk behaviors, providing a reliable assessment of this type of behavior in university student populations. Although it is appropriate to consider incorporating additional sexual risk behaviors as behavioral trends change, the current version of the SRBS may have immediate practical benefits for a population in which barriers to receiving adequate and efficient assessment exist (Cassidy et al., 2018). Furthermore, other existent scales assessing sexual risk behaviors tend to focus on self-confidence to adopt safer sexual behaviors (e.g., Basen-Engquist et al., 2013), providing insight into the antecedents of sexual risk, rather than into actual engagement in this type of behavior. Conversely, the SRBS provides insight into actual behaviors and the frequency in which students engage in them. The SRBS may therefore help ascertain the prevalence of engagement in sexual risk, and when used in longitudinal studies, it may enable researchers to detect changes in students’ sexual behavior over time and under specific psychological conditions.

Although further research will need to further investigate the convergent validity of the SRBS, its properties make it a promising candidate to be used alongside other measures, potentially enabling researchers to shed a light on the relationship between social psychological constructs (e.g., self-efficacy and social norms) and engagement in actual risk behaviors, while retaining clear empirical insight into the prevalence of these behaviors. For instance, it might be useful to examine its correlation with other behaviors known to be associated with sexual risk in other populations, such as “kink” and BDSM (McGregor, 2015). Furthermore, in view of recent research showing the association of identity issues (e.g., threat to one’s sense of self) and specific psychological states such as sexual arousal with engagement in sexual risk behavior, it would be beneficial to examine the relationship of the SRBS with other psychological constructs (see Ariely & Loewenstein, 2006; Jaspal et al., 2021). The SRBS will be especially relevant to the health professions given that it may also enable practitioners to identify a need for student referral to other services, such as counseling and psychotherapy (Mirzaei et al., 2016).

One of the major benefits of the SRBS is its brevity, and the scale has both pragmatic cogency as well as sound psychometric properties. Therefore, the SRBS provides a fast, reliable, and valid screening for key indicators of sexual risk behavior when students arrive at university, and routinely, in their progression through their academic career. For example, academic institutions would benefit from designing surveys to screen those at higher risk of engaging in sexual risk behaviors as efficiently as possible, ideally dedicating no longer than a minute or two to accommodate students’ timing and needs, preventing fatigue, frustration, and unengaged responses in students, without discounting accuracy.

A brief one-factor assessment is also a practical and time-efficient option for busy general practitioners and surgeries to incorporate, because the SRBS does not require additional knowledge of psychometrics and scale administration. Therefore, the SRBS could be used as either an assessment scale to be completed and coded or as a brief self-awareness-raising tool, where patients retain the scale and are encouraged to use it as an aide memoir for risky behavior, alongside clear information on how and where to access care, should they need it. This process could reduce workload for health centers that are not specialist services and protect privacy for the patient who may be reluctant to share their answers with a healthcare professional or general practitioner “on the record” (Jaspal, 2020). It would also empower student patients to take responsibility for their sexual health, helping themselves identify their own risk behavior, considering it personally, and on the “where and how” of relevant specialist services, such as sexual health clinics and/or counseling support.

Some of the existing sexual risk scales are comprehensive though considerably longer than the SRBS and require an understanding of questionnaire administration. Therefore, the SRBS may represent an effective option in first line primary care sexual health interventions, to supplement rather than replace the extant scales. Other scales such as those focusing on perceived confidence in undertaking safer sexual health practice (e.g., Basen-Engquist et al., 2013; Koch et al., 2013), and those measuring engagement with a wider range of sexual risk behaviors (Turchik & Garske, 2009) could then be used to facilitate the assessment of risky behavior in university students, for example via specialist sexual health services, once the initial general risk issues have been identified. Moreover, the metric invariance of SRBS items by gender and by sexual orientation make the SRBS an invariant assessment tool across different groups within the university student populations.

The current study indicated that students who reported high sexual risk were more likely to have received any STI diagnosis in the past 12 months. However, it is acknowledged that this result may be impacted by the opportunistic sampling strategy used, assuming that students who agree to participate in sexual health research are generally more aware of, and concerned about, their risk and health. This limitation is also relevant in view of the relatively low rates of engagement with sexual health services in UK student populations. Nevertheless, if a brief, invariant scale, such as the SRBS, was incorporated into standard health packs on university registration, with an option to submit the data should they wish to, then a balance between sampling bias and a broader reach may be achieved. The likelihood of achieving a more representative sample may also be heightened.

The present study has limitations. First, the SRBS was tested on a sample from a UK student population only, and future research will need to investigate its properties in samples from other populations and contexts, aiming to ascertain its cross-cultural invariance. Second, following the first limitation, some socio-demographic characteristics might be underrepresented, and future research will benefit from testing the properties of the scale using more comprehensive samples. Third, we did not test for the validity of the scale in convergence with other existent scales measuring sexual risk behaviors, and this is a crucial point that must be addressed in future research. Fourth, we did not use a measure of social desirability in association to SRBS scores, and future research would benefit from adding this important analysis to the evaluation of the scale.

## Conclusions

This article summarizes the development, validation, and psychometric testing of the Sexual Risk Behavior Scale (SRBS), which is a novel five-item scale for the measurement of sexual risk behaviors in university student populations. The SRBS exhibits acceptable psychometric properties, reliability, and validity with respect to any STI diagnosis in the past 12 months. As a relatively short and updated tool for measuring sexual risk behaviors in the student population, the SRBS appears to constitute a pragmatic and valid measurement tool for use in both educational and health prevention settings.

## References

[bibr1-01632787211003950] Ariely D. Loewenstein G. (2006). The heat of the moment: The effect of sexual arousal on sexual decision making. Journal of Behavioral Decision Making, 19(2), 87–98. 10.1002/bdm.501

[bibr50-01632787211003950] Bailey J. A. Haggerty K. P. White H. R. Catalano R. F. (2011). Associations between changing developmental contexts and risky sexual behavior in the two years following high school. Archives of Sexual Behavior, 40(5), 951–960. 10.1007/s10508-010-9633-0 20571863 PMC3760485

[bibr51-01632787211003950] Barrett T. S. Brignone E. Laxman D. J. (2020). Furniture: Furniture for Quantitative Scientists (1.9.7) [Computer software]. https://CRAN.R-project.org/package=furniture

[bibr2-01632787211003950] Basen-Engquist K. Mâsse L. C. Coyle K. Kirby D. Parcel G. Banspach S. Nodora J. (2013). Sexual risk behavior beliefs and self-efficacy scales. In Fisher T. D. Clive M. D. Yarber W. L. (Eds.), Handbook of sexuality-related measures (pp. 588–591). Sage. 10.4324/9781315881089-79

[bibr3-01632787211003950] Bender S. S. Fulbright Y. K. (2013). Content analysis: A review of perceived barriers to sexual and reproductive health services by young people. The European Journal of Contraception & Reproductive Health Care, 18(3), 159–167. 10.3109/13625187.2013.776672 23527736

[bibr4-01632787211003950] Bolton P. (2020). Higher education student numbers. The House of Commons Library—UK Parliament. https://commonslibrary.parliament.uk/research-briefings/cbp-7857/

[bibr5-01632787211003950] Brown T. A. (2006). Confirmatory factor analysis for applied research. The Guilford Press.

[bibr52-01632787211003950] Cassidy C. Bishop A. Steenbeek A. Langille D. Martin-Misener R. Curran J. (2018). Barriers and enablers to sexual health service use among university students: A qualitative descriptive study using the Theoretical Domains Framework and COM-B model. BMC Health Services Research, 18. 10.1186/s12913-018-3379-0 PMC6057095 30041649

[bibr6-01632787211003950] Chalmers R. P. (2012). mirt: A Multidimensional Item Response Theory Package for the R Environment. Journal of Statistical Software, 48(1), 1–29. 10.18637/jss.v048.i06

[bibr7-01632787211003950] Chanakira E. O’Cathain A. Goyder E. C. Freeman J. V. (2014). Factors perceived to influence risky sexual behaviours among university students in the United Kingdom: A qualitative telephone interview study. BMC Public Health, 14, 1055. 10.1186/1471-2458-14-1055 25300195 PMC4203964

[bibr8-01632787211003950] Chawla N. Sarkar S. (2019). Defining “high-risk sexual behavior” in the context of substance use. Journal of Psychosexual Health, 1(1), 26–31. 10.1177/2631831818822015

[bibr9-01632787211003950] Choi S. W. Gibbons L. E. Crane P. K. (2011). lordif: An R package for detecting differential item functioning using iterative hybrid ordinal logistic regression/item response theory and monte Carlo simulations. Journal of Statistical Software, 39(8), 1–30. 10.18637/jss.v039.i08f PMC3093114 21572908

[bibr10-01632787211003950] Costello A. B. Osborne J. B. (2005). Best practices in exploratory factor analysis: Four recommendations for getting the most from your analysis. Practical Assessment, 10(7), 1–9.

[bibr11-01632787211003950] De Neef N. Coppens V. Huys W. Morrens M. (2019). Bondage-discipline, dominance-submission and sadomasochism (BDSM). From an integrative biopsychosocial perspective: A systematic review. Sexual Medicine, 7(2), 129–144. 10.1016/j.esxm.2019.02.002 30956128 PMC6525106

[bibr12-01632787211003950] Department of Education. (2019). Participation rates in higher education: Academic years 2006/2007–2015/2016. GOV.UK. https://assets.publishing.service.gov.uk/government/uploads/system/uploads/attachment_data/file/648165/HEIPR_PUBLICATION_2015-16.pdf

[bibr13-01632787211003950] DeVellis R. F. (2016). Scale development: Theory and applications (4th ed.). Sage.

[bibr14-01632787211003950] DilIorio C. Parsons M. Lehr S. Adame D. Carlone J. (1992). Measurement of safe sex behavior in adolescents and young adults. Nursing Research, 41(4), 203–209. 1408860

[bibr15-01632787211003950] Elliot A. J. (2005). A conceptual history of the achievement goal construct. In Elliot A. J. Dweck C. S. (Eds.), Handbook of competence and motivation (pp. 52–72). Guilford.

[bibr16-01632787211003950] Epskamp S. (2015). semPlot: Unified visualizations of structural equation models. Structural Equation Modeling: A Multidisciplinary Journal, 22(3), 474–483. 10.1080/10705511.2014.937847

[bibr17-01632787211003950] Fino E. Giuliani M. Pierleoni L. Gambino G. Cosmi V. Simonelli C. (2018). Factor structure and psychometric properties of the Italian version of the homosexuality scale of the Trueblood sexual attitudes questionnaire. Journal of Homosexuality, 65(6), 784–796. 10.1080/00918369.2017.1364558 28800290

[bibr18-01632787211003950] Fino E. Pierleoni L. Cosmi V. Giuliani M. Gambino G. (2020). Using internet predicts attitudes towards sexual behaviour in Italian psychology students. Sexologies, 29(1), e27–e33. 10.1016/j.sexol.2019.10.001

[bibr19-01632787211003950] Green S. B. Yang Y. (2009). Reliability of summed item scores using structural equation modeling: An alternative to coefficient alpha. Psychometrika, 74(1), 155–167. 10.1007/s11336-008-9099-3

[bibr20-01632787211003950] Horn J. L. (1965). A rationale and test for the number of factors in factor analysis. Psychometrika, 30(2), 179–185. 10.1007/BF02289447 14306381

[bibr21-01632787211003950] Jaspal R. (2019). The social psychology of gay men. Palgrave Pivot. 10.1007/978-3-030-27057-5

[bibr22-01632787211003950] Jaspal R. (2020). Sexual health perceptions among first-year students at a British University. American Journal of Sexuality Education, 15(2), 158–179. 10.1080/15546128.2019.1701597

[bibr23-01632787211003950] Jaspal R. Lopes B. Wignall L. Bloxsom C. (2021). Predicting sexual risk behaviour in British and European Union university students in the United Kingdom. American Journal of Sexuality Education. 10.1080/15546128.2020.1869129

[bibr53-01632787211003950] Jorgensen T. D. Pornprasertmanit S. Schoemann A. M. Rosseel Y. Miller P. Quick C. Garnier-Villarreal M. Selig J. Boulton A. Preacher K. Coffman D. Rhemtulla M. Robitzsch A. Enders C. Arslan R. Clinton B. Panko P. Merkle E. Chesnut S.…Ben-Shachar M. S. (2020). semTools: Useful Tools for Structural Equation Modeling (0.5-3) [Computer software]. https://CRAN.R-project.org/package=semTools

[bibr24-01632787211003950] Karabenick S. A. Woolley M. E. Friedel J. M. Ammon B. V. Blazevski J. Bonney C. R. Groot E. D. Gilbert M. C. Musu L. Kempler T. M. Kelly K. L. (2007). Cognitive processing of self-report items in educational research: Do they think what we mean? Educational Psychologist, 42(3), 139–151. 10.1080/00461520701416231

[bibr25-01632787211003950] Kenny D. A. (2015). Measuring model fit. David A. Kenny. http://davidakenny.net/cm/fit.htm (2015)

[bibr26-01632787211003950] Koch P. B. Colaco C. Porter A. W. (2013). Sexual health practices self-efficacy scale. In Fisher T. D. Davis C. M. Yarber W. L. (Eds.), Handbook of sexuality-related measures (p. 345). Sage.

[bibr27-01632787211003950] Lewis R. Tanton C. Mercer C. H. Mitchell K. R. Palmer M. Macdowall W. Wellings K. (2017). Heterosexual practices among young people in Britain: Evidence from three national surveys of sexual attitudes and lifestyles. Journal of Adolescent Health, 61(6), 694–702. 10.1016/j.jadohealth.2017.07.004 PMC5723633 29169520

[bibr28-01632787211003950] Linacre J. M. (2009). Local independence and residual covariance: A study of Olympic figure skating. Journal of Applied Measurement, 10(2), 1–13. 19564696

[bibr29-01632787211003950] Linacre J. M. Wright B. (1992). Combining (Collapsing) and splitting categories. Measurement Transactions, 6(3), 233–235.

[bibr30-01632787211003950] McDonald R. P. (1999). Test theory: A unified treatment. Taylor & Francis.

[bibr31-01632787211003950] McGregor V. (2015). P14.07 Sexual health promotion and STI prevention on the margins: Kink, BDSM, and sexually adventurous women. Sexually Transmitted Infections, 91, A200–A201. 10.1136/sextrans-2015-052270.519

[bibr32-01632787211003950] Mirzaei M. Ahmadi K. Saadat S. H. Ramezani M. A. (2016). Instruments of high risk sexual behavior assessment: A systematic review. Materia Socio-Medica, 28(1), 46–50. 27047267 10.5455/msm.2016.28.46-50 PMC4789722

[bibr54-01632787211003950] Molinares C. Kolobova I. Knox D. (2017). Anal sexual practices among undergraduate students. Journal of Positive Sexuality, 3, 21–36.

[bibr33-01632787211003950] Moreira Rodrigues L. Dumith Carvalho S. Paludo Dos Santos S. (2018). Condom use in last sexual intercourse among undergraduate students: How many are using them and who are they? Ciencia & Saude Coletiva, 23(4), 1255–1266. 10.1590/1413-81232018234.16492016 29694579

[bibr34-01632787211003950] Patton G. C. Sawyer S. M. Santelli J. S. Ross D. A. Afifi R. Allen N. B. Arora M. Azzopardi P. Baldwin W. Bonell C. Kakuma R. Kennedy E. Mahon J. McGovern T. Mokdad A. H. Patel V. Petroni S. Reavley N. Taiwo K.…Viner R. M. (2016). Our future: A Lancet commission on adolescent health and wellbeing. The Lancet, 387(10036), 2423–2478. 10.1016/S0140-6736(16)00579-1 PMC5832967 27174304

[bibr35-01632787211003950] Pinyaphong J. Srithanaviboonchai K. Chariyalertsak S. Phornphibul P. Tangmunkongvorakul A. Musumari P. M. (2018). Inconsistent condom use among male university students in Northern Thailand. Asia-Pacific Journal of Public Health, 30(2), 147–157. 10.1177/1010539517753931 29409333

[bibr36-01632787211003950] Public Health England. (2018). An STI is diagnosed in a young person every 4 minutes in England. GOV.UK. https://www.gov.uk/government/news/an-sti-is-diagnosed-in-a-young-person-every-4-minutes-in-england

[bibr37-01632787211003950] R Core Team. (2019). R: A language and environment for statistical computing. R Foundation for Statistical Computing.

[bibr38-01632787211003950] Revelle W. (2020). Psych: Procedures for psychological, psychometric, and personality research (1.9.12.31). https://CRAN.R-project.org/package=psych

[bibr39-01632787211003950] Revelle W. Rocklin T. (1979). Very simple structure: An alternative procedure for estimating the optimal number of interpretable factors. Multivariate Behavioral Research, 14(4), 403–414. 10.1207/s15327906mbr1404_2 26804437

[bibr40-01632787211003950] Rizopoulos D. (2006). ltm: An R package for latent variable modeling and item response analysis. Journal of Statistical Software, 17(1), 1–25. 10.18637/jss.v017.i05

[bibr41-01632787211003950] Rosseel Y. (2012). Lavaan: An R package for structural equation modeling. Journal of Statistical Software, 48(2), 1–36. 10.18637/jss.v048.i02

[bibr42-01632787211003950] Samejima F. (1969). Estimation of latent ability using a response pattern of graded scores1. ETS Research Bulletin Series, 1969(1), i–169. 10.1002/j.2333-8504.1968.tb00153.x

[bibr43-01632787211003950] Schmitt T. A. (2011). Current methodological considerations in exploratory and confirmatory factor analysis. Journal of Psychoeducational Assessment, 29(4), 304–321. 10.1177/0734282911406653

[bibr44-01632787211003950] Turchik J. A. Garske J. P. (2009). Measurement of sexual risk taking among college students. Archives of Sexual Behavior, 38(6), 936–948. 10.1007/s10508-008-9388-z 18563548

[bibr45-01632787211003950] van der Linden W. J. Hambleton R. K. (1997). Item response theory: Brief history, common models, and extensions. In van der Linden W. J. Hambleton R. K. (Eds.), Handbook of modern item response theory (pp. 1–28). Springer. 10.1007/978-1-4757-2691-6_1

[bibr46-01632787211003950] Vivancos R. Abubakar I. Hunter P. R. (2008). Sex, drugs and sexually transmitted infections in British university students. International Journal of STD & AIDS, 19(6), 370–377. 10.1258/ijsa.2007.007176 18595873

[bibr47-01632787211003950] Vivancos R. Abubakar I. Hunter P. R. (2010). Foreign travel associated with increased sexual risk-taking, alcohol and drug use among UK university students: A cohort study. International Journal of STD & AIDS, 21(1), 46–51. 10.1258/ijsa.2009.008501 19933205

[bibr48-01632787211003950] White A. Hingson R. (2013). The burden of alcohol use: Excessive alcohol consumption and related consequences among college students. Alcohol Research: Current Reviews, 35(2), 201–218. 24881329 10.35946/arcr.v35.2.11 PMC3908712

